# Hypermobile Disorders and Their Effects on the Hip Joint

**DOI:** 10.3389/fsurg.2021.596971

**Published:** 2021-03-25

**Authors:** Ian M. Clapp, Katlynn M. Paul, Edward C. Beck, Shane J. Nho

**Affiliations:** ^1^Section of Young Adult Hip Surgery, Division of Sports Medicine, Department of Orthopedic Surgery, Rush University Medical Center, Chicago, IL, United States; ^2^Department of Orthopedic Surgery, Wake Forest Baptist Health, Winston-Salem, NC, United States

**Keywords:** hip, femoroacetabular impingement syndrome, hypermobile, hyperlaxity, hypermobility, femoroacetabular impingement

## Abstract

Hypermobility, or joint hyperlaxity, can result from inherited connective tissue disorders or from micro- or macrotrauma to a joint. The supraphysiologic motion of the hip joint results in capsuloligamentous damage, and these patients have a propensity to develop femoroacetabular impingement syndrome (FAIS) and labral injury. In this review, the recent literature evaluating the definitions, history, incidence, genetics, and histology of hypermobile disorders is investigated. We then review the clinical evaluation, natural history, and resulting instability for patients presenting with a hypermobile hip. Lastly, treatment options and outcomes will be highlighted.

## Introduction

Hypermobility is becoming an increasingly recognized source of pain and instability of the hip joint (1–4). The etiology of hypermobility can range from heritable connective tissue disorders to the result of micro- or macrotrauma to the joint. While hypermobility with or without Ehlers–Danlos syndrome is relatively rare in the general population, it is of particular interest to hip arthroscopists due to the propensity of these patients to develop femoroacetabular impingement syndrome (FAIS) and labral injury (5). In addition, these patients may be more likely to have capsular laxity following capsular repair resulting in poor outcomes and complications and possibly requiring revision surgery (6–9). Joint hypermobility can be an especially challenging comorbidity and has been associated with a variety of syndromes that exist on a varying spectrum including hip dysplasia, generalized joint hypermobility (GJH), hypermobile Ehlers–Danlos syndrome (hEDS), and hypermobility spectrum disorder. The purpose of this article is to review the causes of hypermobility, the diagnosis, and to summarize literature on hip hypermobility focusing on surgical treatment options and outcomes for these patients.

## Definitions

Hypermobility, also termed ligamentous laxity, refers to excessive motion of a joint. This can be seen as supraphysiologic motion and often presents without symptoms (3). The hip joint capsule acts as a static stabilizer and is comprised of four ligaments: the iliofemoral, pubofemoral, ischiofemoral, and zona orbicularis. In the native hip, laxity of these ligaments can result in pain and microinstability of the joint (2). However, it must be noted that a joint may be hypermobile yet stable, and the differentiating factor between hypermobility and instability is the presence of symptoms (9). When a majority of an individual's synovial joints are capable of excessive motion, the patient is diagnosed with generalized joint hypermobility (GJH) (10), which is a hallmark of hereditary disorders of connective tissue.

The Ehlers–Danlos syndromes (EDSs) are a group of heritable connective tissue disorders characterized by abnormal collagen synthesis, which can affect skin, ligaments, blood vessels, and other organs, often causing articular hypermobility (11, 12). Hypermobile EDS (hEDS) is the most common subtype, often resulting in chronic joint pain and frequent dislocations due to joint hyperlaxity, which can negatively affect a patient's quality of life (13, 14). Prior to 2017, it had been proposed that asymptomatic joint hypermobility and joint hypermobility syndrome (JHS) lay at opposite sides of the same spectrum of disorders and that JHS and hEDS may be equivalent disorders (15–17). Per the 2017 International Criteria for Ehlers–Danlos syndrome, JHS is now referred to as hypermobility spectrum disorder (18), which has been proposed as joint hypermobility plus one or more of its secondary manifestations but not satisfying the criteria for any EDS variant (16).

## History and Incidence

EDS was first studied and classified in the late 1960s, which resulted in the Berlin nosology in 1986 (19). This was the first attempt to categorize and formalize the nomenclature of the different subtypes of the syndrome. Due to newly noticed clinical and molecular variants, a revised classification, the Villefranche nosology, was proposed a decade later and delineated six subtypes (20). The most current nomenclature and classification system came in 2017 with the International Criteria for Ehlers–Danlos syndrome, which changed the naming of joint hypermobility syndrome to hypermobility spectrum disorder (18).

Naal et al. found the prevalence of GJH to be 33% in a cohort of 55 patients with FAIS (21) compared to 3% in normal adult population (22–24). The prevalence of joint hypermobility in the general population is quite low; however, the combined incidence of hypermobility spectrum disorder and hEDS is thought to be 10 in 5,000 (25, 26). GJH is more prevalent in children and adolescence, but this may be attributed to decreases in ranges of motion as age increases (27). It also affects women, Asians, and West Africans more frequently (28, 29).

## Genetics and Histological Findings

There is a lack of a well-defined biologic marker for GJH and hypermobility spectrum disorder. In the case of hEDS, an autosomal dominant inheritance pattern with variable penetrance has been elucidated (17). Monozygotic twins have been shown to have higher concordance rates of joint hypermobility compared to dizygotic twins (60 vs. 36%), suggesting a strong genetic trait that is most likely multifactorial and heterogenous (30, 31). Recently, new genes coding for LZTS1 (32) and Tenascin X protein—a protein that plays an important role in organizing and maintaining the structure of connective tissues (33, 34)—were associated with the hEDS phenotype. However, the exact physiological process remains unknown, and heterozygous TNXB deficiency accounts for a small percentage of hEDS. A genetic mutation seen in a family with hEDS suggests there is overlap with the mutation caused by COL34A, which is normally observed in vascular EDS. This results in intracellular retention of type III collagen (35).

While it has been previously shown that collagen fibril structures are abnormal in patients with hEDS, the etiology is not exactly known (11). The weakened collagen fibers affect the elastic fibers of the skin and the longevity and integrity of ligaments, causing the clinical symptom of hypermobility (36, 37).

## Clinical Evaluation and Criteria for Diagnosis

Evaluation of hip hypermobility should follow the typical sequence of history, physical exam, and imaging workup. Patients presenting with ligamentous laxity may describe mechanical symptoms or apprehension in certain positions (3, 38). A thorough assessment of any prior hip surgery with operative data should be performed, as capsular insufficiency is a leading indication for revision hip arthroscopy (7). Due to the hereditary causes of hypermobility, a detailed family and medical history should be taken. Patients with hEDS often have a history of joint dislocations and extra-articular manifestations such as widespread pain and skin hyperlaxity (39, 40). A thorough physical exam is critical in these patients and should include assessment of the Beighton criteria, posterior impingement with extension, the hip dial test, and the axial distraction test (3, 41).

The Beighton score is used to assess GJH and can help distinguish normal laxity from hypermobility (20). It is a nine-point objective scale, and a patient scoring > 4 indicates the presence of hypermobility (42–45). The maneuvers performed and scoring are located in Table 1. For diagnosis of hypermobility spectrum disorder and hEDS, the Brighton criteria are used. In order to meet the diagnosis, patients must meet the criteria listed in Table 2 (29, 46). Hakim and Grahame (47) developed a validated five-point questionnaire with a sensitivity of 84% and specificity of 85%. Individuals answering yes to two or more of these questions suggests hypermobility: “Can you now (or could you ever) place hands flat on floor without bending knees?”, “Can you now (or could you ever) bend your thumb to touch your forearm?”, “As a child, did you amuse your friends by contorting your body into strange shapes or could you do the splits?”, “As a child or teenager, did your kneecap or shoulder dislocate on more than one occasion?”, and “Do you consider yourself ‘double-jointed’?”.

**Table 1:** Beighton score for hypermobility.

**Maneuver**	**Scoring**	**Example**
Passive dorsiflexion of the fifth finger >90 degrees with forearm flat	1 point for each side (Maximum Score of 2)	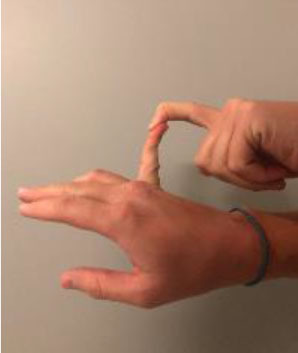
Passive apposition of the thumb to the flexor aspect of the forearm	1 point for each side (Maximum Score of 2)	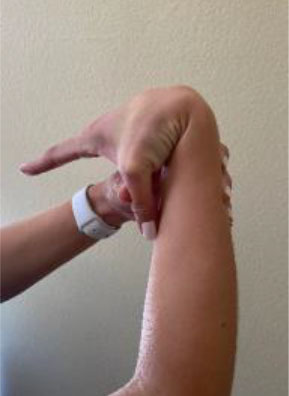
Hyperextension of elbow >10 degrees	1 point for each side (Maximum Score of 2)	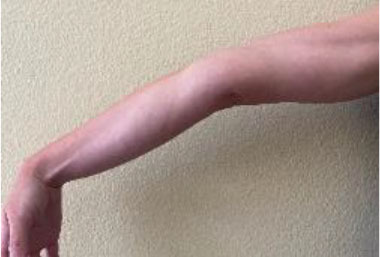
Hyperextensibility of the knee >10 degrees	1 point for each side (Maximum Score of 2)	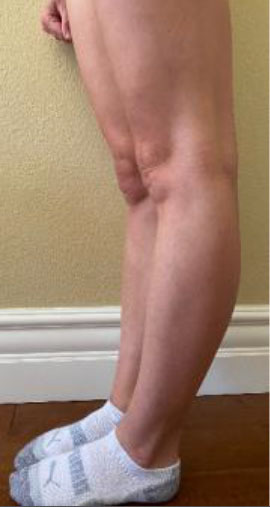
Flexion of waist with palms on the floor (and with the knees fully extended)	1 point (Maximum Score of 1)	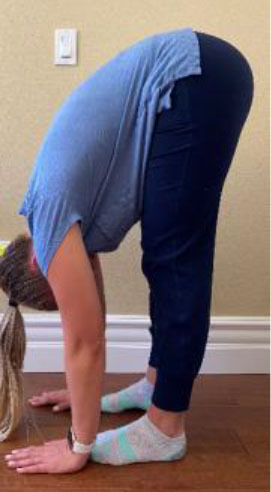
Total Score	Maximum Score of 9	

*Scores for each maneuver are totaled with a maximum score of 9 and a score > 4 indicating hypermobility. The Beighton score is a major criterion for the Brighton Criteria*.

**Table 2:** Brighton criteria.

**Major criteria**	**Description**
Beighton score > 4	
Polyarthralgias	>3 months in four or more joints
**Minor criteria**	
Beighton score <4	1–3 if younger than 50-years old, 0–3 if older than 50-years
Oligoarthalgias	Arthralgia >3 months in 1–3 joints or back pain, spondylolisthesis, spondylolysis, or spondylosis
Dislocation or subluxation	More than one joint, more than one occasion
Soft tissue lesions	>3 lesions (e.g., epicondylitis, tenosynovitis, bursitis)
Marfanoid habitus	Arachnodactyly, ratio of arm span to height >1.03, ratio of upper segment to lower segment <0.89
Skin abnormalities	Hyperextensibility, striae, thin skin, abnormal scarring
Eye signs	Drooping eyelids, myopia, or antimongoloid slant
Varicose veins, hernia, or uterine/rectal prolapse	
Mitral valve prolapse	
**Exclusions**	
Presence of Marfan syndrome	
Presence of EDS	Other than hEDS

*Hypermobility is suggested in the presence of two major criteria, one major and two minor criteria, four minor criteria, or an unequivocally affected first-degree relative in the family*.

*EDS, Ehlers–Danlos syndrome. hEDS, hypermobile Ehlers–Danlos syndrome*.

The imaging workup of these patients should include standard hip radiographs [anteroposterior pelvis (AP), false profile view, and Dunn lateral] as well as the splits radiograph. The splits radiograph consistently shows lateral femoral head translation and creation of a vacuum sign (9). The AP film should be examined for the femoral head cliff sign (48), which has been associated with an intraoperative diagnosis of microinstability. The Femoro-Epiphyseal Acetabular Roof (FEAR) index must also be measured to assess for instability (49). Lastly, magnetic resonance imaging can be utilized to better assess the labrum and capsuloligamentous structures.

## Natural History and Instability of Hip Hypermobility

Whether hypermobility is acquired or inherited, it presents with capsuloligamentous laxity and can lead to instability and possibly recurrent subluxations and repeated dislocations of the hip joint (5, 50). Acquired hypermobility may result from local micro- or macrotrauma (frank dislocation or injury). Repetitive movements in extreme ranges of motion may cause compensatory soft tissue laxity, which may be desired in some athletes (51). Untreated, these patients may develop recurrent soft tissue injuries and chronic pain, and hypermobile patients may be more prone to developing premature arthritis and capsular degeneration (15, 52, 53).

Hypermobility may confer a competitive advantage in athletes participating in dance, gymnastics, or cheer where flexibility and extreme ranges of motion are necessary to compete at high levels (1, 5, 54). However, these biomechanics and repetitive loading can do damage to the cartilage and result in instability of the joint (55). Those with hypermobility are at an increased risk for injury and suffer a longer recovery time (5, 56). Furthermore, placing the hip at extreme, supraphysiologic ranges of motion is thought to predispose these patients to developing impingement (5). Extreme ranges of motion can place the hip in a potentially impinging or unstable positions and make the joint more susceptible to impingement-induced instability in which the anterior cam impingement creates a fulcrum, resulting in posterolateral instability of the femoral head (57). Charbonnier et al. revealed that significant subluxation of the femoral head was present at extremes of motion and directly correlated with impingement when using MRI in ballet dancers (58). This finding was further supported by Wassilew et al., who found high rates of posterior subluxation in positions of impingement (59). These findings suggest that hypermobility in the presence of FAIS can be a predisposing factor for hip instability.

Hip microinstability is a relatively new concept characterized by hip hypermobility in the setting of hip pain or dysfunction (60, 61) and is difficult to diagnose, with no objective criteria for diagnosis (62). Numerous etiologies have been described, and microtrauma in the setting of osseous and soft tissue abnormalities may contribute to the development (3, 62). As prior studies have identified the hip capsule as a major stabilizing structure, ligamentous laxity is also a contributing factor to microinstability (63). In a cadaveric study, hip capsular laxity caused increased joint rotations, femoral head translations, and abnormal movement of the femoral head, leading to microinstability (2). This excess motion of the femur relative the acetabulum can lead to damage to the labrum, cartilage, and capsular structures over time (10). Additionally, Devitt et al. demonstrated that in patients undergoing hip arthroscopy for the treatment of FAIS, the presence of GJH was predictive of hip capsular thickness, with those with GJH having a thinner hip capsule (<10 mm) than those without (64).

## Treatment and Outcomes

Management of hypermobility includes both operative and non-operative treatment. Patients with hypermobility disorders should implement lifestyle changes as well as enroll in an exercise program (29). Physical therapy programs should be individualized to consider the patients' condition and focus on strengthening the dynamic musculature surrounding the hip in order to increase stability (62, 65).

For hypermobile patients with refractory symptoms of pain or instability, open or arthroscopic surgery may be indicated. Surgical treatment options are directed toward correcting the underlying pathologic etiology such as FAIS or a labral tear, with proper capsular management being integral to successful outcomes and prevention of postoperative instability in these patients (3, 66). Capsular closure is necessary, with capsular plication or capsular shift often being used for patients with connective tissue disease and hypermobility (6, 67, 68). In a cadaveric study performed by Waterman et al. (69), the intracapsular volume of a native hip joint, capsular plication of T-capsulotomy, and capsular shift of the interporal capsulotomy were compared. The authors demonstrated significant reduction in intra-articular volume of the hip undergoing capsular plication of the T-capsulotomy and capsular shift of the interporal capsulotomy when compared to the native hip. Furthermore, in cases of extreme ligamentous insufficiency, capsular reconstruction may be utilized using an iliofemoral ligament reconstruction with an Achilles tendon allograft (70).

Biomechanical studies have demonstrated that capsulotomy size inversely affects the force required for hip distraction and increases hip movement, leading to instability (71, 72). Additionally, capsular defects have been reported following capsulotomy during hip arthroscopy, with capsular insufficiency being a leading indication for revision hip arthroscopy (7). In a systematic review, ligamentous laxity was cited as a possible risk factor for post-arthroscopy dislocation, as it was cited in 11.1% of cases of dislocation (8). Given the inclination of patients with GJH, hypermobility disorder spectrum, and hEDS to have capsular laxity, capsular management is essential for these patients in order to restore sufficient stability.

There is a paucity of literature on surgical outcomes of hypermobile patients. However, available studies demonstrate improvement in patient-reported outcomes and favorable results. Kalisvaart and Safran examined 32 patients with hip instability treated with capsular plication and found significant improvements in the modified Harris Hip Score (mHHS) and the International Hip Outcome Tool (iHOT) score at a minimum of 12 months postoperatively (73). They also demonstrated a high level of return to sport, with 9 out of 11 collegiate or professional athletes returning. Arthroscopic hip surgery has been shown to be effective at treating soft tissue hip instability caused by hEDS. In 16 hips with hEDS, Larson et al. reported significant improvements for the mHHS, 12-Item Short Form Health Survey (SF-12), and VAS pain score at a mean of 45 months (6). This cohort underwent meticulous capsular plication and did not suffer any iatrogenic dislocations postoperatively.

Stone et al. compared outcomes of female patients with and without GJH and demonstrated no significant difference between the groups in terms of postoperative range of motion, pain, and functional outcomes at 2-year follow-up (74). Ukwuani et al. examined return to sport in dancers following hip arthroscopy, with 33% of the patient cohort meeting the diagnosis of GJH (43). It was shown that hypermobility did not affect 2-year postoperative functional outcome scores or return to dancing activity. These encouraging results are supported by two recent studies. Maldonado et al. performed a matched cohort analysis and exhibited that patients with ligamentous laxity had no significant difference in mHHS, Non-arthritic Hip Score, Hip Outcome Score—Sports Specific Scale, and VAS pain at 2-year follow-up (67). Moreover, these patients achieved minimal clinically important difference and patient acceptable symptomatic state at rates comparable to patients without hypermobility. In a cohort of 63 competitive dancers, there was significant improvement in mHHS and Hip disability and Osteoarthritis Outcome Scores at 3-years postoperatively, with 84% of dancers returning to sport (4).

These outcomes support that hip arthroscopy with correct capsular management is a highly effective treatment for patients with hip pathology with concurrent hypermobility. While the short- to mid-term follow-up support improved outcomes, larger studies with long-term outcomes are needed.

## Conclusions

Hypermobility refers to the excessive range of motion of a joint and can result from hereditary connective tissue disorders or repetitive local trauma. Regardless of the etiology, hip capsular laxity can lead to instability, pain, and dysfunction, often requiring treatment. Hip arthroscopy with proper capsular management such as capsular plication or shift is an effective treatment for stabilization and produces favorable outcomes in this patient group. Further research is needed to clarify long-term outcomes and treatment modalities to reduce instability in these patients.

## Author Contributions

All authors had significant contributions to manuscript preparation and editing.

## Conflict of Interest

The authors declare that the research was conducted in the absence of any commercial or financial relationships that could be construed as a potential conflict of interest.
